# Overall and progression-free survival with cabazitaxel in metastatic castration-resistant prostate cancer in routine clinical practice: the FUJI cohort

**DOI:** 10.1038/s41416-019-0611-6

**Published:** 2019-11-13

**Authors:** Magali Rouyer, Stéphane Oudard, Florence Joly, Karim Fizazi, Florence Tubach, Jérémy Jove, Clémentine Lacueille, Stéphanie Lamarque, Estelle Guiard, Aurélie Balestra, Cécile Droz-Perroteau, Annie Fourrier-Reglat, Nicholas Moore, on behalf of the FUJI Investigators, Samir Abdiche, Elisabeth Angellier, Dominique Beal-Ardisson, Guillaume Bera, Jean-François Berdah, Olivier Bernard, Anne-Sophie Blanc, Isabelle Bonnet, Florence Borde, Mathieu Bosset, Fabien Brocard, Sylvie Cailleres, Damien Carlier, Elisabeth Carola, Christine Clippe, Corinne Dagada, Ariane Darut-Jouve, Jérôme Dauba, Christine Dolmazon, Patrick Dube, Mounira El Demery, Philippe Evon, François Guichard, Ali Hasbini, Benjamin Hoch, Ludmila Hu, Brigitte Laguerre, Hortense Laharie, Nathalie Lemoine, Nadia Levasseur, Christophe Louvet, Delia Molnar, Isabelle Moullet, Sophie Nahon, Stéphane Oudard, Jean-Briac Prevost, Frank Priou, Mansour Rastkhah, Stéphane Remy, Jean-Louis Reynoard, Hamida Talbi, Youssef Tazi, Erika Viel, Stéphane Vignot, Laurent Vives

**Affiliations:** 1 Bordeaux PharmacoEpi, CIC1401, University of Bordeaux, Bordeaux, France; 2 Medical Oncology Department, Hôpital Européen Georges Pompidou, Université Paris Descartes, Paris, France; 3 Medical Oncology Department, Centre François Baclesse, CHU Côte de Nacre, Caen, France; 4 Medical Oncology Department, Institut Gustave Roussy, Villejuif, France; 5 Sorbonne Université, AP-HP, Hôpital Pitié-Salpêtrière, Département Biostatistique Santé Publique et Information Médicale, Unité de Recherche Clinique PSL-CFX, Centre de Pharmacoépidémiologie (Cephepi), Inserm, UMR 1123 ECEVE, CIC-1421, Paris, France; 6 Inserm U1219, Bordeaux, France; 7 Hôpital R. Boulin, Libourne, France; 8 CH Layne, Mont-de-Marsan, France; 9 Hôpital privé J. Mermoz, Lyon, France; 10 CH Bretagne Sud, Lorient, France; 11 Hôpital privé Toulon-Hyères Ste Marguerite, Hyères, France; 12 Centre Radiothérapie Oncologie Moyenne Garonne, Agen, France; 13 Centre Marie Curie, Arras, France; 14 CH Jean Bernard, Valenciennes, France; 15 CH de Saintonge, Saintes, France; 16 Hôpital privé Drôme-Ardèche, Guilherand-Granges, France; 17 Centre oncologie de Gentilly, Nancy, France; 18 CH du Pays d’Aix, Aix-en-Provence, France; 19 Centre Léonard de Vinci, Dechy, France; 20 Groupe Hospitalier Public Sud Oise, Senlis, France; 21 CH de Romans, Romans-sur-Isère, France; 22 CH de Pau, Pau, France; 23 Centre d’oncologie du Parc-polyclinique Drevon, Dijon, France; 24 CH Layne, Mont-de-Marsan, France; 25 Infirmerie Protestante de Lyon, Caluire-et-Cuire, France; 26 Clinique de l’Europe, Amiens, France; 27 Clinique du Cap d’Or, La Seyne-sur-Mer, France; 28 CH de Bar-le-Duc, Bar-le-Duc, France; 29 Polyclinique Bordeaux Nord, Bordeaux, France; 30 Clinique Pasteur, Brest, France; 31 Centre Azuréen de Cancérologie, Mougins, France; 32 Hôpital Ste Camille, Bry-sur-Marne, France; 33 Centre Eugène Marquis, Rennes, France; 34 Clinique Tivoli, Bordeaux, France; 35 Hôpital privé La Louvière, Lille, France; 36 CH de Cahors, Cahors, France; 37 Institut Mutualiste Montsouris, Paris, France; 38 CH de Brive, Brive-la-Gaillarde, France; 39 Clinique de la Sauvegarde, Lyon, France; 40 CH du Pays d’Aix, Aix-en-Provence, France; 41 Hôpital européen Georges Pompidou, Paris, France; 42 Radiopole Artois, Arras, France; 43 CH départemental de Vendée, La Roche-sur-Yon, France; 44 Centre Praz-Coutant, Passy, France; 45 Centre d’oncologie et radiothérapie, Bayonne, France; 46 Centre clinical, Soyaux, France; 47 Hôpital Saint Camille, Bry-sur-Marne, France; 48 Strasbourg Oncologie Libérale, Strasbourg, France; 49 Cabinet d’oncologie médicale, Chalon-sur-Saône, France; 50 Hôpital Louis Pasteur, Le Coudray, France; 51 CH Comminges Pyrénées, Saint-Gaudens, France

**Keywords:** Chemotherapy, Prostate cancer, Outcomes research

## Abstract

**Background:**

Cabazitaxel is a treatment of metastatic castration-resistant prostate cancer (mCRPC) after docetaxel failure. The FUJI cohort aimed to confirm the real-life overall and progression-free survival (OS, PFS) and safety of cabazitaxel.

**Methods:**

Multicentre, non-interventional cohort of French mCRPC patients initiating cabazitaxel between 2013 and 2015, followed 18 months.

**Results:**

Four hundred one patients were recruited in 42 centres. At inclusion, median age was 70, main metastatic sites were bones (87%), lymph nodes (42%) and visceral (20%). 18% had cabazitaxel in 2nd-line treatment, 39% in 3rd-line and 43% in 4th-line or beyond. All had prior docetaxel, and 82% prior abiraterone, enzalutamide or both. Median duration of cabazitaxel treatment was 3.4 months. Median OS from cabazitaxel initiation was 11.9 months [95% CI: 10.1–12.9]. In multivariate analyses, grade ≥ 3 adverse events, visceral metastases, polymedication, and >5 bone metastases were associated with a shorter OS. Main grade ≥ 3 adverse events were haematological with 8% febrile neutropenia.

**Conclusion:**

Real-life survival with cabazitaxel in FUJI was shorter than in TROPIC (pivotal trial, median OS 15.1 months) or PROSELICA (clinical trial 20 vs 25 mg/m^2^, median OS, respectively, 13.4 and 14.5 months). There was no effect of treatment-line on survival. No unexpected adverse concerns were identified.

**Study registration:**

It was registered with the European Medicines Agency EUPASS registry, available at www.encepp.eu, as EUPAS10391. It has been approved as an ENCEPP SEAL study.

## Background

Prostate cancer is the second most common cancer in men and the fifth most common cause of cancer death worldwide.^1^ In France, prostate cancer ranks first in incidence in men (incidence rate 187 per 100,000), and third in mortality (18 per 100,000) in 2012.^2^ Thanks to screening resulting in early identification, prostate cancer is frequently diagnosed at an early stage, when it can be cured by radical prostatectomy or radiation therapy. Around 10% of patients are diagnosed at a metastatic stage with usually a very poor prognosis.^3^ Androgen deprivation therapy (ADT) with anti-androgens or LH-RH derivatives has been shown to delay progression. However, over time most prostate cancers will acquire resistance to ADT. This is referred to as castration-resistant prostate cancer (CRPC). In addition, to the patients diagnosed at the metastatic stage most CRPC become metastatic (mCRPC).^4^

Treatment options for mCRPC have long been limited,^5^ with only mitoxantrone with prednisone being licensed for its palliative effect without survival benefit. In 2004, docetaxel in combination with prednisone was shown to improve overall survival (OS).^6^ Over the last decade, three additional treatments have been licensed for treatment of mCRPC following failure of docetaxel, the new generation taxane cabazitaxel^7^ and two androgen-receptor-targeted therapies, abiraterone acetate^8^ and enzalutamide,^9^ with similar results.^10^ The latter two agents have subsequently been licensed also for first-line treatment of mCRPC.

Cabazitaxel was approved as second-line treatment based on the results of the TROPIC trial, which enrolled 755 mCRPC patients progressing during or after docetaxel treatment.^11^ Cabazitaxel plus prednisone demonstrated a significant OS improvement compared to mitoxantrone plus prednisone (hazard ratio: 0.70 [95% confidence interval: 0.59–0.83]). The recommended dose of cabazitaxel is 25 mg/m² administered as a one-hour intravenous infusion every three weeks in combination with oral prednisone or prednisolone 10 mg administered daily throughout treatment.

Since then, the PROSELICA clinical trial has confirmed the non-inferiority of cabazitaxel 20 mg/m^2^ (C20) versus 25 mg/m² (C25) in 1200 post docetaxel mCRPC patients. Median OS, time to PSA progression and PSA response rate were 14.5 months, 6.8 months and 42.9%, for C25, respectively, and 13.4 months, 5.7 months and 29.5% for C20.^12^

At the time of the marketing authorisation of cabazitaxel in France in October 2011, the French Health Authorities requested a post-marketing study documenting the effectiveness and safety of cabazitaxel in everyday clinical practice. The FUJI study was designed to meet this request. The primary objective of FUJI was to evaluate OS in mCRPC patients treated by cabazitaxel in daily practice. Secondary objectives included PSA response, progression-free survival, profile of patients receiving cabazitaxel including analgesic use and safety of cabazitaxel.

## Methods

### Study design

FUJI (Follow-Up of Jevtana^®^ in real life) is a French multicentre non-interventional cohort study of patients with mCRPC starting treatment with cabazitaxel between 1 September 2013 and 31 August 2015 and followed 18 months.

### Participants

The methodology was the same as in real-life studies of other cancer treatments:^13–15^ in France, cabazitaxel is only available through hospital prescriptions on a named-patient basis. All hospitals dispensing cabazitaxel were identified between September 2013 and December 2014 from sales data provided by the manufacturer. The pharmacists of these centres were invited to participate in the study. Those who accepted provided a list and the contact details of the oncologists who had prescribed cabazitaxel during the study period. These oncologists were invited to participate in the study and if so, include in the study all patients who had received at least one cycle cabazitaxel, irrespective of the number of cycles received, except those who had been enrolled in a clinical trial. The patients who were enrolled by the oncologists were compared to dispensing records by the pharmacists to ensure full consecutive enrolment. If alive at the time of data compilation, patients were asked to confirm their non-opposition to the use of data.

Enrolment continued until the target sample size of 400 patients had been achieved.

### Data collection

Clinical and prostate specific antigen (PSA) data were extracted from the hospital records during the eighteen-month period following the first administration of cabazitaxel, or until the patient died and entered into an electronic case report form by a dedicated clinical research assistant. All data collected were validated by the participating physician and included the following: date of first cabazitaxel administration, patient demographics, disease history including prior treatments, cabazitaxel treatment modalities, outcomes (clinical, biological, radiological), date of death, analgesic use and adverse events (AEs) reported during cabazitaxel treatment. Adverse events were coded using the current MedDRA classification and their severity was coded according to the grading system of the National Cancer Institute’s Common Terminology for the Classification of Adverse Events (NCI-CTCAE v4.0). Adverse events requiring hospital admission were also identified.

### Study end-points

The primary end-point was OS over eighteen months, calculated from first cabazitaxel administration. Secondary end-points were PSA response (defined by a PSA decrease of at least 50% from baseline) and progression-free survival (PFS) defined as in TROPIC^16^ (PSA and/or radiological and/or clinical progression or death). PSA progression was defined as an increase in PSA of at least 25% and of at least 2 ng/ml compared to the lowest post-cabazitaxel treatment value (PSA nadir), confirmed by a 2nd PSA value at least 3 weeks later. Radiological progression was defined by RECIST criteria version 1.0 as per physician judgment.^17^ Clinical progression was based on pain/symptoms and analgesic consumption as per physician judgment.

### Statistical analysis

The target sample size (400 subjects) was chosen to estimate OS at eighteen months with a precision of 4.9%, considering a median OS in TROPIC trial of 15.1 months.^16^ OS and PFS were estimated using Kaplan-Meier survival analysis. Cox proportional hazard models over the eighteen-month follow-up period were used to identify risk factors associated with death or progression. The variables entered into the Cox models are listed in supplementary data Table 1, and pertain to factors occurring before cabazitaxel, to patient status at first cabazitaxel injection and to per-treatment events. Survival estimates were also provided for patients with synchronous or metachronous metastases.

All statistical analyses were performed with SAS^®^ (SAS Institute, version 9.4, Cary, USA), according to a statistical analysis plan defined prior to data lock.

### Ethics

The study was conducted in accordance with all relevant national legislation and guidelines for observational studies, with approval from the French national data-protection Agency (CNIL). The study protocol was submitted to and approved by the French health authorities as part of the post-marketing commitments of the manufacturer of cabazitaxel (Sanofi-Aventis). All patient data in the study database were anonymised. The study was registered with the EU PAS registry (ENCEPP/SDPP/10391).

## Results

### Study population

**Fig. 1:**
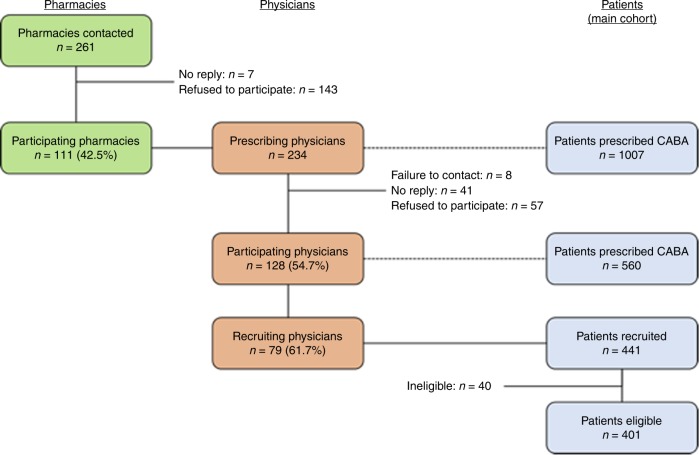
Patient flowchart (CABA: cabazitaxel)

Overall, 261 hospital pharmacies that had dispensed cabazitaxel at least once between September 2013 and December 2014 were contacted and invited to participate in the study. Of these, 93 pharmacies (35.6% of those contacted) accepted to participate and provided contact details of 234 physicians who had prescribed cabazitaxel. One hundred twenty eight of these agreed to participate, and 79 had recruited patients into the study when the target patient sample size was met. When recruitment was stopped, 401 patients had been recruited, between September 2013 and August 2015, in 42 centres. The patient recruitment process is illustrated in Fig. 1.

Patient characteristics are presented in Table 1. At cabazitaxel initiation, median age was 70 years median time elapsed since prostate cancer diagnosis was 5.5 years, 20% had visceral metastases and 15.7% had an ECOG PS ≥ 2. At cancer diagnosis, median Gleason score was 7. About 40% had synchronous metastases. All patients had been treated with docetaxel before cabazitaxel. Prior to cabazitaxel initiation, 18.0% had received only one life-extending therapy (LET) line for mCRPC (docetaxel), 38.7% two previous LET lines, 22.7% 3 previous LET lines and 20.7% 4 or 5 previous LET lines. Most patients (81.5%) had been treated previously by novel androgen-receptor (AR)-targeted agents (abiraterone acetate, enzalutamide or both). The majority of patients (*n* = 389; 97.0%) had associated comorbidities, notably cardiovascular (69.2%), digestive (46.0%), endocrine (44.5%), urogenital (39.3%), musculoskeletal (33.4%) and respiratory (30.1%) disorders.

**Table 1:** Patient characteristics at inclusion

	*n* = 401
*Disease history*	
Gleason score at initial diagnosis of prostate cancer (*n*; %)	
Missing data	18 (4.5)
4–5	9 (2.2)
6–7	186 (46.4)
8–10	188 (46,9)
Onset of metastases (*n*; %)	
Synchronous	161 (40.1%)
Metachronous	237 (59.1%)
Time from primary diagnosis to metastasis (months, median [IQR])	22.8 [0.4–75.3]
*Patient characteristics at cabazitaxel initiation*	
Median age (years, median [IQR])	70.0 [65–77]
ECOG score (*n*; %)	
Not available	237 (59.1%)
0–1	101 (25.2%)
≥2	63 (15.7%)
Visceral metastases (*n*; %)	79 (19.7%)
>5 bone metastases (*n*; %)	269 (67.1%)
PSA value (ng/ml, median [IQR])	112.5 [38–380]
Polymedication, >5 drugs (excluding cancer treatments) (*n*; %)	83 (20.7%)
Number of prior life-extending therapies (*n*; %)	
1 treatment (Docetaxel)	72 (18.0%)
2 treatments	155 (38.7%)
3 treatments	91 (22.7%)
4 or 5 treatments	83 (20.7%)
Docetaxel before cabazitaxel initiation (*n*; %)	401 (100%)
Abiraterone acetate before cabazitaxel initiation (*n*; %)	307 (76.6%)
Enzalutamide before cabazitaxel initiation (*n*; %)	134 (33.4%)
Abiraterone acetate and/or enzalutamide before cabazitaxel initiation (*n*; %)	327 (81.5%)

### Treatment with cabazitaxel

Cabazitaxel was most often administered every three weeks (*n* = 364, 90.8%) at a starting dose of 25 mg/m^2^ (*n* = 184, 50.5%). Other administration schedules were: <25 mg/m² every 3 weeks (*n* = 160, 44.0%), >25 mg/m² every 3 weeks (*n* = 13, 3.6%), 12–17 mg/m² every 2 weeks (*n* = 36, 9.2%). In 125 patients (31.2%), the dose was reduced over the course of treatment.

The median duration of cabazitaxel treatment was 3.4 months with a median of five cycles. Among patients who had discontinued cabazitaxel at 18 months (95%), the main reasons for discontinuation were disease progression or disease-related death (83.2%), or the occurrence of AEs (15.2%).

### Survival outcome and response rates

**Fig. 2:**
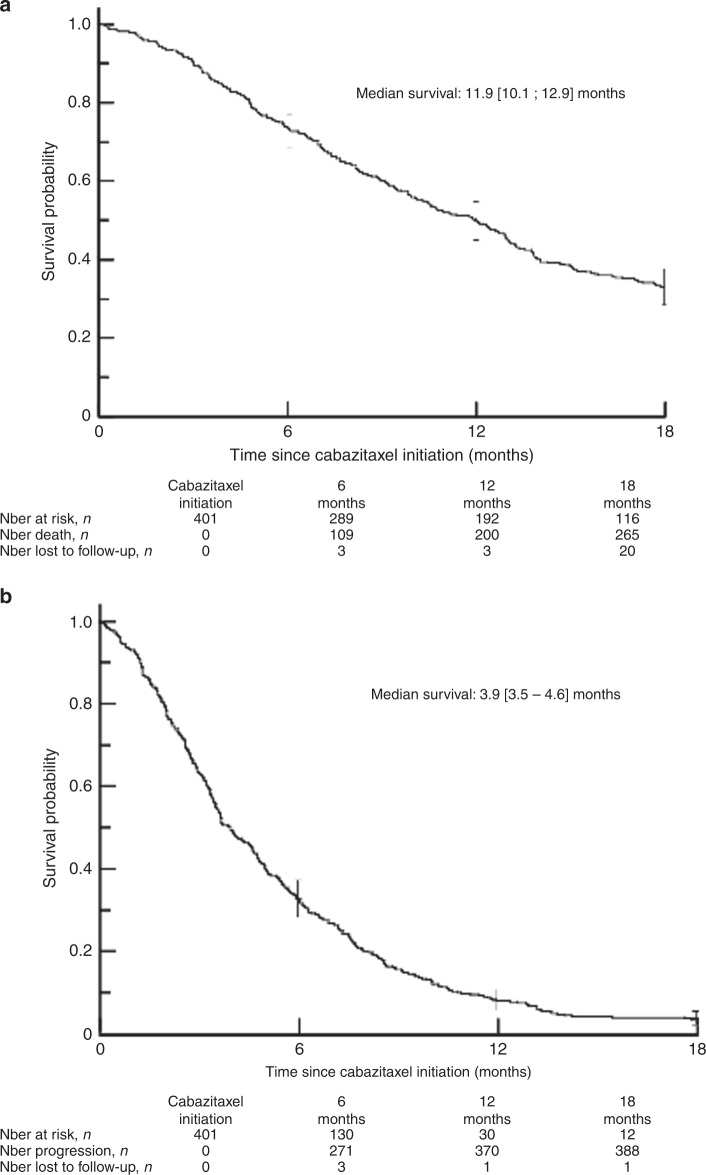
Overall and progression-free survival. **a** Overall survival. **b** Progression-free survival

The 18-month OS rate was 32.4% [95% CI: 27.8–37.1] with a median OS of 11.9 months [10.1–12.9]. The Kaplan-Meier survival curve is presented in Fig. 2a. In multivariate Cox analysis, factors independently associated with a shorter OS were related to pre-treatment variables: <10 years since PC diagnosis, hazard ratio (HR), 95% confidence interval (95% CI) 1.52 [1.04–2.17], progression during docetaxel treatment (1.69 [1.13–2.53]) or within 3 months after the last docetaxel injection (1.51 [1.07–2.14]), when the patient had received two or more Life-extending treatments before cabazitaxel (1.39 [1.00–1.92]), when the interval between the last docetaxel administration and first cabazitaxel injection was less than 6 months (1.41 [1.03–1.92]); to patient status at first use of cabazitaxel: presence of visceral metastases (1.98 [1.40–2.80]) or more than five bone metastases (1.74[1.20–2.53]), plasma prostate specific antigen > 135 ng/mL (1.36 [1.01–1.82]) or when there were more than 5 concomitant non-cancer treatments at first cabazitaxel injection (1.74 [1.23–2.45]). Finally, overall survival was shorter when there was at least one grade ≥ 3 adverse event during cabazitaxel treatment (2.05 [1.53–2.73]).

In the pre-specified subgroup analyses, median OS from first cabazitaxel administration ranged from 9.9 months [6.6–12.9] for patients with one previous LET line, 12.1 months [8.5–15.0] for patients with two previous LET lines, 12.9 months [10.0–14.7] for patients with three previous LET lines and 11.7 months [8.1–13.5] for patients with four previous LET lines before cabazitaxel initiation (see supplementary data e-Fig. 1). No significant differences were observed in OS between patients with synchronous or metachronous metastases or in patients having previously been treated with different disease-modifying cancer treatments.

### Progression-free survival

The median PFS was 3.9 months [95% CI: 3.5–4.6] (Fig. 2b). The PFS rate was 32.4% [27.9–37.0] at 6 months and 3.1% [1.7–5.1] at 18 months. In the multivariate Cox analysis, factors independently associated with a shorter PFS at 6 months were intensification of analgesic use to level III (HR = 2.31 [1.70–3.14]), disease progression within 3 months of the last docetaxel administration and before cabazitaxel initiation (HR = 2.30 [1.68–3.15]), and at least one AE of grade ≥ 3 during cabazitaxel treatment (HR = 1.50 [1.14–1.97]). No differences in PFS were observed between the pre-specified subgroups.

The overall response rate varied from 11.0% (radiological response) to 38.9% (PSA response) depending on the criterion used (Table 2). However, regardless of the criterion, <3% of patients achieved a complete response.

**Table 2:** Response rates at any time during follow-up

	PSA response	Radiological response	Clinical response
Not available	5 (1.2%)	2 (0.5%)	17 (4.2%)
Not evaluable	49 (12.2%)	129 (32.2%)	51 (12.7%)
Complete response	Not applicable	None	6 (1.5% [0.5–3.3])
Partial response	146 (36.4% [31.7–41.1])	44 (11.0% [7.9–14.0])	73 (18.2% [14.4–22.0])
Stable disease	92 (22.9% [18.8–27.1])	94 (23.4% [19.3–27.6])	177 (44.1% [39.3–49.0])
Progression	99 (24.7% [20.5–28.9])	132 (32.9% [28.3–37.5])	77 (19.2% [15.3–23.1])
Overall response rate^a^	156 (38.9% [34.1–43.7])	44 (11.0% [7.9–14.0])	79 (19.7% [15.8–23.6])
Medical benefit^b^	248 (61.8% [57.1–66.6])	138 (34.4% [29.8–39.1])	256 (63.8% [59.1–68.5])

Data are presented as frequency counts (%), with their 95% confidence intervals if appropriate, for the 401 evaluable patients

^a^Overall response rate: complete response + partial response

^b^Medical benefit: complete response + partial response + stable disease

**Fig. 3:**
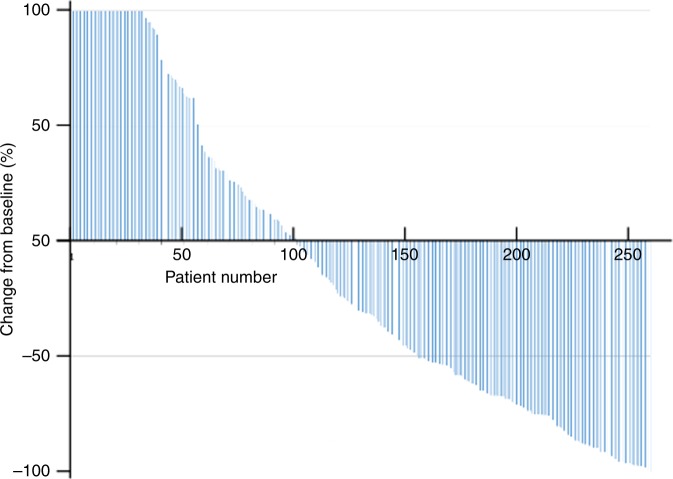
Change in PSA concentrations during cabazitaxel treatment from pre-treatment values. Each line represents the on-treatment change in PSA in an individual patient, ranked from the largest increase (cropped at 100%) to the largest decrease. An increase of more than 100% in best PSA response concerned 33 patients of the 258 evaluable patients

PSA response was available for 258 patients (64.3%) (Fig. 3). In 103 patients (39.9%), PSA decreased by ≥50%, with a median time to reach this threshold of 4.1 months. The median time to PSA progression was 5.0 months.

### Adverse events

Almost all patients (99.0%) experienced at least one AE during cabazitaxel treatment AEs requiring hospitalisation occurred in 41.1% of patients; the first AE occurred during the first or second cabazitaxel administration for half of patients (52.1%). At least one AE of Grade ≥ 3 was observed for 55.4% of patients, and these required hospitalisation in 26.6%.

There was no difference in the event rates for grade ≥ 3 AE according to the number of previous treatment lines, from 54.2% with just one previous line, to 45.8% with four or five treatment lines before cabazitaxel.

Adverse events are presented by System-Organ Class (SOC) and Preferred Term (PT) in Table 3. The most frequent AEs were haematological, in particular anaemia (92.5%), thrombocytopaenia (28.9%) and neutropaenia (26.9%). Other frequent AEs were fatigue and asthenia (69.6%), diarrhoea (39.9%), nausea (29.9%). GCSF was systematically used before each cabazitaxel infusion in 45.9% of patients. This was 45.8% when there was one treatment line before cabazitaxel, 54.8% for two treatment lines, 31.9 % for three treatment lines, and 44.6% for four or five treatment lines before cabazitaxel.

**Table 3:** Adverse events occurring during cabazitaxel treatment, by system-organ class and preferred term

FUJI cohort	*n* = 401	
Adverse event (AE)	All grades	Grade ≥ 3
≥1 AE, *n* (%)	397 (99.0%)	222 (55.4%)
Haematological AE, *n* (%)	371 (92.5%)	160 (39.9%)
Anaemia	363 (90.5%)	108 (26.9%)
Thrombocytopenia	116 (28.9%)	21 (5.2%)
Neutropenia	108 (26.9%)	60 (15.0%)
Leukopenia	100 (24.9%)	38 (9.5%)
Febrile neutropenia	32 (8.0%)	32 (8.0%)
General disorders, *n* (%)	331 (82.5%)	17 (4.2%)
Fatigue and asthenia	279 (69.6%)	13 (3.2%)
Gastrointestinal disorders, *n* (%)	274 (68.3%)	17 (4.2%)
Diarrhoea	160 (39.9%)	10 (2.5%)
Nausea	120 (29.9%)	4 (1.0%)
Vomiting	79 (19.7%)	5 (1.2%)
Renal and urinary disorders, *n* (%)	152 (37.9%)	37 (9.2%)
Haematuria	81 (20.2%)	6 (1.5%)
Renal failure	30 (7.5%)	29 (7.2%)
Urinary retention	24 (6.0%)	2 (0.5%)
Infections and infestations, *n* (%)	124 (30.9%)	20 (5.0%)
Septicaemia and septic shock	20 (5.0%)	20 (5.0%)

The most frequent grade ≥ 3 AEs were anaemia (26.9%), neutropenia (15.0%) including febrile (8%), leukopenia (9.5%), renal failure (7.2%), thrombocytopenia (5.2%) and septicaemia and septic shock (5.0%). Six cabazitaxel-related deaths occurred, five of which were related to sepsis or septic shock with febrile neutropenia. These patients had at least one G-CSF treatment before or during the cycle when febrile neutropenia occurred, except for one patient who had an infectious shock after the first cabazitaxel administration.

### Analgesic use

Use of Level I analgesics was more common during cabazitaxel treatment (70.3% of patients) than before (44.9%) or after (58.6%). Of the patients who were taking analgesics at cabazitaxel initiation, 30.9% reduced their analgesic consumption during the follow-up period. Analgesic consumption increased in 41.2% of patients. After discontinuation of cabazitaxel, the frequency of analgesic use remained stable in 67%. These data are summarised in supplementary data Table 2.

## Discussion

The objective of this study was to evaluate the effectiveness and safety of cabazitaxel in the treatment of mCRPC patients in everyday clinical practice in France. In this heavily treated population (39% received cabazitaxel in 3rd line, 23% in 4th line, 21% in 5th line or beyond), the rate of OS at 18 months after cabazitaxel initiation was 32.4%. The most frequent AEs reported were haematological reactions, with 8% febrile neutropenia.

The strengths of this study were the relatively large sample size (*n* = 401), the broad eligibility criteria that encompassed all mCRPC patients treated with cabazitaxel whatever the treatment line, and the requirement that participating physicians include all their patients treated with cabazitaxel during the study recruitment period. In addition, follow-up was complete, and the status of all patients could be assessed at eighteen months. These features ensured that outcome could be determined with precision and be representative of outcome of mCRPC patients treated with cabazitaxel in everyday oncology practice in France. Nonetheless, some selection bias may be inherent to the voluntary participation of pharmacists and investigators. Around half the hospital pharmacies and around half the identified prescribers refused to participate in the study, so it is not certain that management of patients in these centres that did not participate was comparable to that observed in this study. Nonetheless, a previous study evaluating the impact of prescriber participation found no difference in patient outcomes between participating and non-participating physicians.^18^

The median OS was shorter here (11.9 months [10.1–12.9] than in the Phase 3 TROPIC trial,^11^ (15.1 months [14.1–16.3])) or in the PROSELICA trial (Median OS 13.4 and 14.5 months for the initial cabazitaxel doses of 20 and 25 mg/m^2^).^12^ There are two potential explanations for these differences. First, patients included in FUJI were in general older and more fragile than those enrolled in the clinical trials, where severe eligibility criteria were applied. For example, patients with poor performance status (ECOG score > 2) or with severe haematological, hepatic, renal or cardiac comorbidities were excluded in clinical trials. In contrast, eligibility criteria in FUJI were broad and all mCRPC patients treated by each participating investigator over the study period were enrolled, whatever their health status and whatever the duration of treatment. Indeed, if TROPIC inclusion and exclusion criteria were applied to FUJI, only two of 401 patients (0.5%) would have been eligible. Also, in TROPIC, all patients received cabazitaxel in second-line after docetaxel. In contrast with TROPIC, at the time of FUJI abiraterone acetate and enzalutamide were both available and only 18% received cabazitaxel immediately after docetaxel, with a median OS of 9.9 months. A possible explanation for the shorter OS as compared to TROPIC (15.1 months) may be that such patients might have had aggressive clinical features thought unlikely to respond to new AR-targeted agents. A phase 2 randomised controlled trial has indeed shown that taxanes were more effective than novel AR-targeted agents (abiraterone acetate or enzalutamide) in poor-prognosis mCRPC patients.^19^ Another explanation may be that patients with a very compromised status and short expected survival are often not included in clinical trials, whereas they are part of real-life observational studies.

In FUJI, median OS with cabazitaxel in fourth line setting or beyond after novel AR-targeted agents was 12.9 months [10.0–14.7] indicating that cabazitaxel retains its activity after these patients. In the multivariate analysis, multiple previous treatment lines and a high burden of comorbidity (as witnessed by the number of concomitant medications being taken), were independent predictors of mortality.

Effectiveness data on cabazitaxel in everyday oncology practice have also been published from the compassionate use programs established in the Netherlands,^20^ Korea^21^ and Germany^22^ before cabazitaxel was commercially available. In the study from the Netherlands, median OS was 8.7 months [IQR: 6.0–15.9], which is close to the value observed in the FUJI study and again shorter than that reported in TROPIC. On the other hand, the Korean study reported a longer median OS (16.5 months [95% CI: 12.1–20.9]), close to the value reported in TROPIC. The German study reported mean OS (13.9 months [range: 0.7–35.8]) rather than median OS and is thus not really comparable. The publications on the Italian^23^ and Spanish^24^ compassionate use programs did not present data on survival.

The safety profile of cabazitaxel in FUJI was essentially similar to that observed in TROPIC or PROSELICA, and no unanticipated safety issues arose. The proportion of patients presenting AEs of severity Grade 3 or 4 was 55.4% in FUJI compared to 57.4% in TROPIC^11^ and 39.7% (20 mg/m^2^) or 54.5% (25 mg/m^2^) in PROSELICA.^12^ In all studies, haematological AEs were the most common. Although anaemia was reported at a relatively similar frequency in both studies (90% in FUJI and 97% in TROPIC), the reporting frequency of neutropenia was much lower in FUJI (26.9%) than in TROPIC (94%) or PROSELICA (41.8% and 73.3%). This may be explained by less intensive biological monitoring in real-life practice than in TROPIC, in which neutrophils were systematically measured at nadir, 8–10 days after each administration of cabazitaxel and G-CSF more systematically given.^25^ The frequency of febrile neutropenia was identical in both studies (8%), compared to 2.1 and 9.6% for C20 and C25, respectively, in PROSELICA.^12^ Six cabazitaxel-related deaths due to sepsis or septic shock, with febrile neutropenia in five, were reported in the FUJI study (1.5%), emphasising the importance of using prophylactic G-CSF from cycle 1 as per EORTC guidelines and carefully monitoring neutrophil counts in patients receiving cabazitaxel. In the PROSELICA C20 and C25 treatment groups, 2.1% and 3.2% of patients, respectively, died within 30 days of the last dose of cabazitaxel as a result of AEs.^12^ The occurrence of AEs was not a major reason for stopping treatment with cabazitaxel, concerning only around one in eight patients. The safety profile of cabazitaxel in FUJI can also be compared with the safety data collected in the European compassionate use program for cabazitaxel, which enrolled 746 patients.^26^ The frequency of adverse event reporting was generally higher in the compassionate use program than in the FUJI study, except for neutropenia. The compassionate use program reported 17.0% of patients with Grade 3 neutropenia, 5.5% with febrile neutropenia, 1.3% with neutropenic sepsis and seven deaths related to neutropenia or its complications.

Cabazitaxel was generally used as recommended in the prescribing information at the time of the study. The licensed indication (mCRPC, after docetaxel) was respected in all patients. Although the recommended treatment regimen of one cycle every three weeks was followed by most patients (90.8%), the recommended starting dose of 25 mg/m² was used in only half of the patients. Although dose reductions are recommended in patients with hepatic failure, the number of patients with hepatic disease (*n* = 47) could not account for the large number of patients starting at the lower dose of 20 mg/m² (196 patients). The fact that patients were less fit than in TROPIC (only 0.5% satisfied inclusion/exclusion criteria of TROPIC), may have contributed to it. The PROSELICA Phase 3 clinical trial^12^ has also demonstrated that a lower starting dose of 20 mg/m² was non-inferior to the approved dose of 25 mg/m² with a lower incidence of AEs. Prescribers may have been aware of that study and anticipated its results.^27^

In conclusion, this real-life cohort study of cabazitaxel in mCRPC patients in France demonstrates that median OS at 18-months is slightly lower in everyday oncology practice than what was reported in the pivotal clinical trial, due to the presence of features of poor prognosis at baseline and use of cabazitaxel in 3rd line or beyond in 82% of patients. There were no unexpected safety issues, with severe neutropenia being the most important risk to consider when prescribing cabazitaxel.

## Supplementary information

supplemental material

## Data Availability

The data of this study are available, as per the ENCEPP code of conduct available at www.encepp.eu, upon providing the reasons for the request and the proposed study protocol.
