# Supplementary material for: Overall and progression-free survival with cabazitaxel in metastatic castration-resistant prostate cancer in routine clinical practice: the FUJI cohort
Source: Br J Cancer. 2019 Nov 13;121(12):1001–8. doi: 10.1038/s41416-019-0611-6 (PMC6964680; doi:10.1038/s41416-019-0611-6)
Supplement: Supplementary file 1 — supplemental material [file 41416_2019_611_MOESM1_ESM.docx]

Supplementary data

e-Table 1. Variables included in the Cox analysis

| Age at inclusion |
| --- |
| Number of previous chemotherapy lines before initiation of cabazitaxel |
| Time between last administration of docetaxel and first administration of cabazitaxel |
| Time to progression after last docetaxel administration |
| PSA concentrations at initiation of cabazitaxel |
| Gleason score at cancer diagnosis |
| Time elapsed since prostate cancer diagnosis |
| Number of bone metastases at initiation of cabazitaxel |
| Presence of visceral metastases at initiation of cabazitaxel |
| Type of metastases (synchronous or metachronous) |
| Polymedication > 5 drugs (other than cancer treatments) |
| Use of other life-prolonging treatments (abiraterone acetate, enzalutamide or radium^223^) |
| Change in analgesic consumption during cabazitaxel treatment |
| At least one Grade ≥ 3 AE during cabazitatel treatment |
| Reduction of cabazitaxel dose during follow-up |
| Delay of cabazitaxel administration during follow-up |

e-Table 2. Analgesic use

|  | Before cabazitaxel initiation | During cabazitaxel treatment | After cabazitaxel discontinuation |
| --- | --- | --- | --- |
| Any analgesic use | 180 (44.9%) | 282 (70.3%) | 235 (58.6%) |
| Level I (NSAIDS) | 13 (7.2%) | 39 (13.8%) | 27 (11.5%) |
| Level I (other) | 82 (45.6%) | 200 (70.9%) | 149 (63.4%) |
| Level II | 51 (28.3%) | 103 (36.5%) | 83 (35.3%) |
| Level III | 116 (64.4%) | 177 (62.8%) | 193 (82.1%) |
| *Change of level of treatment intensity during treatment* | | | |
| Any decrease |  | 124 (30.9%) |  |
| Any increase |  | 165 (41.2%) |  |
| Decrease only |  | 29 (7.2%) |  |
| Increase only |  | 70 (17.5%) |  |
| Decrease and increase |  | 95 (23.7%) |  |
| *Introduction of a higher-level analgesic* | | | |
| Level II |  | 31 (7.7%) |  |
| Level III |  | 68 (17.0%) |  |

Data are presented as frequency counts for the 401 evaluable patients.

e-Figure 1

e-Figure 1: overall survival as a function of the number of previous treatment lines.
